# POWER OF SOCIAL SUPPORT AGAINST DEPRESSIVE SYMPTOMS OF OLDER AFRICAN AMERICANS

**DOI:** 10.1093/geroni/igz038.580

**Published:** 2019-11-08

**Authors:** Seungjong Cho

**Affiliations:** Case Western Reserve University, Cleveland, Ohio, United States

## Abstract

Depressive symptomatology is one of the most prevalent mental health problems. About 20% of Americans experience depressive symptoms in their lives (Gotlib & Hammen, 2014). Social support, on the other hand, was proved to be a powerful buffer against depressive symptoms of older adults (Kim & Ross, 2009; Sheiman & Meersman, 2004). Few studies have explored this association exclusively among older African Americans who had a culture of powerful social support. Therefore, the purpose of this study is to compare the effects of different sources of social support (from spouse/partner, children, relatives, and friends) against depressive symptoms among older African Americans. This study analyzed the 2014 Health and Retirement Study (HRS; N = 187). Depressive symptomatology was operationalized as a count outcome (number of having symptoms; CES-D8 scale). A negative binomial regression model of depressive symptoms showed that higher levels of spousal support were associated with lower levels of depressive symptoms (coefficient = –.179, p < .001). For each additional score in spousal support, the expected log count of the number of depressive symptoms was decreased by .179. Other sources of social support were not significant predictors of depressive symptoms among older African Americans in this study. Among the covariates, self-rated health (coefficient = .358, p < .001) and household income (logged; coefficient = –.275, p = .014) were significant. The current study supported the results of previous studies showing the power of positive spousal interactions against depressive symptomatology, especially among a nationally representative sample of older African Americans.

